# Cervical Cancer Cells with Positive Sox2 Expression Exhibit the Properties of Cancer Stem Cells

**DOI:** 10.1371/journal.pone.0087092

**Published:** 2014-01-28

**Authors:** Xiao-Fang Liu, Wen-Ting Yang, Rui Xu, Jun-Tian Liu, Peng-Sheng Zheng

**Affiliations:** 1 Department of Reproductive Medicine, the First Affiliated Hospital, Xi'an Jiaotong University Medical School, Xi’an, The People’s Republic of China; 2 Department of Pharmacology, Xi'an Jiaotong University Medical School, Xi’an, The People’s Republic of China; 3 Department of Biochemistry and Molecular Biology, Xi'an Jiaotong University Medical School, Xi’an, The People’s Republic of China; 4 Division of Cancer Stem Cell Research, Key Laboratory of Environment and Genes Related to Diseases, Ministry of Education, Xi’an Jiaotong University Medical School, Xi’an, The People’s Republic of China; University of Quebec at Trois-Rivieres, Canada

## Abstract

**Background:**

Although Sox2 expression has been found in several types of cancer, it has not yet been used to identify or isolate CSCs in somatic carcinoma.

**Methods:**

SiHa and C33A cells stably transfected with a plasmid containing human Sox2 transcriptional elements driving the enhanced green fluorescent protein (EGFP) reporter were sorted into the Sox2-positive and the Sox2-negative populations by FACS, and Sox2 expression was detected by western blot and immunohistochemistry. The differentiation, self-renewal and tumor formation abilities, as well as the expression of the stemness and the EMT related genes of the Sox2-positive and the Sox2-negative cervical cancer cells were characterized *in vitro* and *in vivo*.

**Results:**

A pSox2/EGFP system was used to separate the Sox2-positive and the Sox2-negative cells from cervical cancer cell lines, SiHa and C33A cells. Compared with the Sox2-negative cells, the Sox2-positive SiHa and C33A cells exhibited greater capacities for self-renewal, differentiation and tumor formation. Furthermore, Sox2-positive SiHa and C33A cells expressed higher levels of stemness-related genes, such as Sox2/Bmi-1/Oct4/ALDH1, and EMT-related genes, such as vimentin/snail/β-catenin. Taken together, all these results indicated that cells expressing endogenous Sox2 are CSCs in cervical carcinomas.

**Conclusion:**

This study is the first to establish a functional link between endogenous Sox2 expression and CSCs in cervical carcinomas. Additionally, this study demonstrated that it is feasible to develop a tool to isolate CSCs from somatic tumors based on the expression of the endogenous nuclear protein Sox2 instead of cell surface markers.

## Introduction

The cancer stem cell (CSC) hypothesis suggests that tumor masses may arise from a single cancer cell with stem-like properties. These CSCs are postulated to have the capacities of self-renewal and differentiation, and they can regenerate the tumor mass and all tumor cell types found within. Experimental evidence supporting the hypothesis was first generated in 1997 by Dick’s group, who demonstrated that human leukemia is driven by a small population of leukemic stem cells capable of transferring the disease to NOD/SCID mice [1]. This concept was extended to solid tumors by Clarke and Wicha, who demonstrated that human breast cancer contains a subpopulation of cells with stem-like properties bearing the surface markers CD44^+^/CD24^−^/lin^−^ [2]. Subsequently, CSCs have been identified and prospectively isolated from a variety of malignancies, including brain cancers [3], prostate cancer [4], melanoma [5], multiple myeloma [6], colon cancer [7], [8], pancreatic cancer [9] and head and neck cancers [10]. However, CSCs have not been identified in cervical carcinomas.

Most cancer stem cell assays have thus far depended on a variety of different cell surface markers, including CD133, CD44, CD166, and CD24. Using these surface markers, CSCs from primary tumor tissues can be enriched using FACS technology, and their propagation can be tested in immunodeficient mice. However, surface markers can only be used to isolate the most common CSCs, and these markers are often unstable in many somatic cancers [11]. CSCs enriched from primary cultures based on serial xenograft passage *in vivo* have been reported to contain an inconsistent subpopulation after isolation using the surface markers CD133 and CD44 [12]. Additionally, the results obtained with CSCs isolated using the same surface marker are not consistent among laboratories. Thus, it is becoming necessary to search for cytoplasmic or nuclear makers that can be used for the isolation of CSCs [13].

In a previous study, we identified the expression of the embryonic stem cell-specific transcription factor Sox2 in primary cervical cancer tissues and tumorspheres formed by primary cervical carcinoma cells, and we found that Sox2 functions as an oncogene in cervical carcinogenesis by promoting cell growth and tumorigenicity [14], [15]. Our results suggest that Sox2 may be a potential marker for cervical CSCs. Additionally, Sox2 controls the pluripotency, self-renewal and proliferation of embryonic stem cells. It has been shown that murine and human embryonic stem cells and neural stem cells have high Sox2 activity [16], [17], [18], and increased Sox2 expression has also been found in breast and glioblastoma CSC populations [19], [20]. Taken together, these data imply that Sox2 is a candidate nuclear marker for CSCs.

In the present study, we stably transfected two cervical cancer cell lines, SiHa and C33A, with a plasmid containing the human Sox2 transcriptional elements driving EGFP expression. We demonstrated that Sox2-positive cervical cancer cells shared all the characteristics of CSCs.

## Materials and Methods

### Cell Lines and Culture Conditions

The human cervical cancer cell lines SiHa, HeLa, C33A, and CaSki were all purchased from the American Type Culture Collection (ATCC; Manassas, VA). SiHa, HeLa, and C33A cells were maintained in Dulbecco’s Modified Eagle’s Medium (DMEM; Sigma-Aldrich, St Louis, MO) supplemented with 10% heat-inactivated fetal bovine serum (FBS; Invitrogen, Carlsbad, CA). CaSki cells were cultured in McCoy’s 5A medium (Sigma-Aldrich) with 10% FBS.

### Construction of pSox2/EGFP

The ∼11.5 kb human Sox2 promoter was amplified by polymerase chain reaction (PCR) from SiHa genomic DNA with the following primers: forward, 5′–gctagcgaccacatctggctgcttgtatatttaac-’3 and reverse, 5′-catgcggggcgctgtgcgcg-’3. Additionally, the 3' untranslated region (3'UTR), poly (A) tail, and 3′ enhancer of Sox2 were also amplified by PCR with the following primers: forward, 5′-tgagggccggacagcgaac-’3 and reverse, 5′-gtcgacatgagaggtgagtgcagtgcaattac-’3. The vector sequence of interest, including the independent SV40 promoter-driven neomycin resistance cassette, and the EGFP sequence were also amplified from the pIRES2-EGFP vector (Invitrogen). Subsequently, these fragments were cloned into TOPO vectors (Invitrogen), and the accuracy of the DNA sequence was confirmed by sequencing. The correct human Sox2 promoter, UTR/enhancer, EGFP, and vector were subsequently cloned using an In-Fusion PCR Cloning Kit, and the resulting vector was designated phSox2/EGFP (Takara Bio Inc, Dalian, China).

### Immunohistochemistry and Immunocytochemistry

Immunohistochemistry was performed on 4-µm sections of paraffin-embedded tissues. Tumor tissue sections were successively deparaffinized and rehydrated prior to pretreatment with 10 mM sodium citrate antigen retrieval buffer (pH 6.0) in a steam pressure cooker. After treating with 3% H_2_O_2_, the following antibodies were incubated with the sections overnight at 4°C: anti-Sox2 (1∶100), anti-Ki67 (1∶500), anti-ALDH1 (BD Biosciences, 1∶50), anti-Bmi1 (1∶100), anti-Oct4 (1∶100), anti-Nanog (1∶100), anti-Ki67 (1∶80), anti-vimentin (1∶200), anti-snail (1∶150), anti-β-catenin (1∶250), and anti-E-cadherin (1∶200). All antibodies were obtained from Santa Cruz Biotechnology (Santa Cruz, CA) unless otherwise specified. The tissue sections were then incubated with biotinylated immunoglobulin G (IgG) for 30 minutes at room temperature. After washing, the sections were incubated in streptavidin-peroxidase complex for 30 minutes, and immunostaining was performed using 0.05% 3′-diaminobenzidine followed by counterstaining with hematoxylin. Sera from non-immunized goats or mice were used as negative controls.

Additionally, cells were cultured on glass coverslips for 48 hours, fixed with 4% paraformaldehyde for 20 minutes, and permeabilized with 0.3% Triton X-100 for 20 minutes at room temperature. The expression levels of the different proteins in these cells were determined by immunocytochemistry as described above.

### TUNEL Assay

Paraffin-embedded tissue slides were prepared from the xenograft tumors. TUNEL staining was detected by the TUNEL assay kit (Roche) according to the manufacturer’s instruction. Apoptotic nuclei were analyzed by counting the total number of TUNEL-positive nuclei, excluding cells undergoing mitosis in 10 random fields.

### Western Blotting

Cell lysates were separated by 10% sodium dodecyl sulfate (SDS) polyacrylamide gel electrophoresis and transferred onto polyvinylidene fluoride (PVDF) membranes. After blocking with 5% fat-free milk in Tris-buffered saline, the following antibodies were used for western blotting: anti-Sox2 (1∶500), anti-ALDH1 (BD Biosciences, 1∶500), anti-Bmi1 (1∶500), anti-Oct4 (1∶500), anti-Nanog (1∶500), anti-vimentin (1∶500), anti-snail (1∶500), anti-β-catenin (1∶500), anti-E-cadherin (1∶500), and anti-β-actin (1∶1000) overnight at 4°C. All antibodies were obtained from Santa Cruz Biotechnology unless otherwise specified. After washing, the bound antibodies were visualized using horseradish peroxidase-conjugated anti-goat, ant-rabbit, or anti-mouse IgG (Thermo Fisher Scientific Inc., New York, NY) and the Immobilon Western Chemiluminescent HRP Substrate (Millipore, Billerica, MA) and subsequently visualized on X-ray films [21].

### Flow Cytometry and Separation of EGFP^+^ and EGFP^−^ Populations by FACS

Cervical cancer cell lines were cultured for 48 hours after transfection with pSox2/EGFP. The cells were digested and resuspended in PBS supplemented with 2% FBS, and the percentage of EGFP^+^ cells was determined using a FACSCalibur flow cytometer (Becton Dickinson, Franklin Lakes, NJ).

Cell cycle analysis was performed using FACS according to the manufacturer’s protocol. The cells were harvested and fixed in 70% ethanol overnight at 4°C. Thirty minutes before FACS analysis, the cells were treated with RNaseA and subsequently stained with propidium iodide (PI, Sigma-Aldrich). Cell cycle distribution was analyzed with a FACSCalibur flow cytometer using Mod-Fit LT software.

To obtain the EGFP^+^ and EGFP^−^ populations, SiHa and C33A cells were transfected with pSox2/EGFP plasmid using Lipofectamine 2000 (Invitrogen). Selection was performed using standard culture medium with 1 mg/mL G418. The generation of single cell-derived cultures was performed using a FACSAria (Becton Dickinson, Franklin Lakes, NJ). The sorting gates were established as the highest and lowest 10% of the EGFP-expressing cells. The cells were cultured in DMEM/F12 with 1x B-27 serum-free supplement (Invitrogen), 20 ng/mL epidermal growth factor [22], and basic fibroblast growth factor (PeproTech Inc., Rocky Hill, NJ).

### Tumorsphere Formation Assay

The sorted cells were maintained in stem cell media consisting of DMEM/F12 basal media, N2 and B27 supplements (Invitrogen), 20 ng/mL human recombinant epidermal growth factor (EGF) and 20 ng/mL basic fibroblastic growth factor (bFGF; PeproTech Inc., Rocky Hill, NJ).

For the tumorsphere formation assay, cells were plated at a density of 200 cells/well in 24-well ultra-low attachment plates or at a density of 1 cell/well in 96-well plates and maintained in stem cell media. Tumorspheres that arose within 2 weeks were recorded. For serial tumorsphere formation assays, the spheres were harvested, disaggregated with 0.25% trypsin/EDTA, filtered through a 40 µm mesh and re-plated as described above.

### Real-time PCR

Total RNA was extracted from the lowest and highest 10% of EGFP-expressing cells with TRIzol reagent (Invitrogen). The RNA concentration was determined, and total cDNA was used as a template for PCR amplification. Real-time quantitative PCR was performed in triplicate for each primer set using an iQ5 Multicolor Real-Time PCR Detection System (Bio-Rad, Hercules, CA). The protocol for real-time PCR was 1 cycle of 95°C for 30 seconds followed by 40 cycles of 95°C for 5 seconds and 60°C for 30 seconds with a subsequent dissociation stage. The cycle threshold value was determined as the point at which the fluorescence exceeded a preset limit determined by the instrument’s software.

### Tumor Xenograft Assay

Six-week-old female NOD/SCID mice were used to assess stem cell properties *in vivo*. Cells were injected into the subcutis on the dorsum with a mixture of 1∶1 Matrigel. The tumor volume (V) was determined by the length (a) and width (b) as V = ab^2^/2. The experimental protocols were evaluated and approved by the Animal Care and Use Committee of the Medical School of Xi’an Jiaotong University.

### Cell Growth and Cell Viability Assays

Cells (2×10^4^) were cultured in triplicate in 35-mm culture dishes for 7 days. The cells were harvested longitudinally and counted using a hemocytometer and a light microscope. Cell viability was assessed using 3-(4,5-dimethylthiazole-yl)-2,5-diphenyl tetrazolium bromide (MTT, Sigma-Aldrich) dye according to a standard protocol. The number of viable cells was determined by measuring the absorbance at 490 nm.

### Statistical Analysis

Statistical analysis was performed with SPSS 16.0 software (SPSS Inc., Chicago, IL). The two-tailed chi-square test was used to determine the significance of differences between covariates. Student’s t test was used to determine statistical significance when two groups were compared. To examine the relationship between two quantitative variables, Pearson’s linear regression analysis was performed. In all tests, p<0.05 was considered statistically significant.

## Results

### Development of the EGFP-expressing Plasmid Driven by Human Sox2 Transcriptional Elements

**Figure 1:**
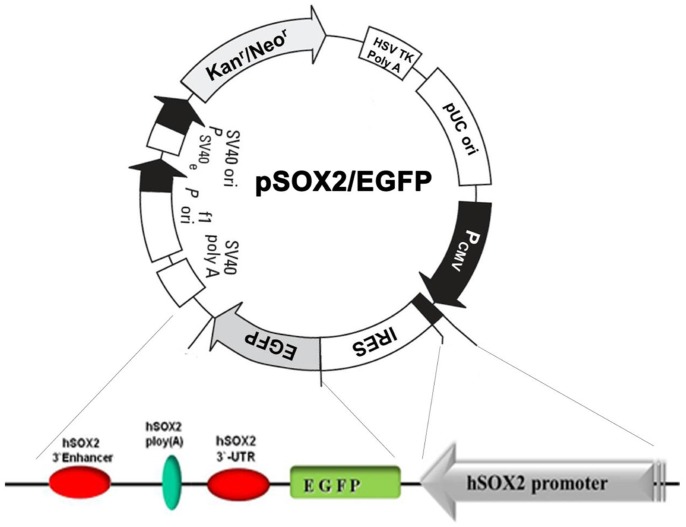
pSox2/EGFP reporter system construction. Schematic of the pSox2/EGFP reporter system. The hSox2 promoter and transcriptional elements including the 3'UTR, poly (A) tail, and 3′ enhancer were cloned into the pEGFP vector.

To isolate the distinct endogenous Sox2-expressing subpopulation and determine whether it has the characteristics of CSCs, we constructed the plasmid pSox2/EGFP, which contains 11.5 kb of the human Sox2 promoter sequence positioned upstream of the EGFP reporter. This plasmid also contains the human Sox2 3′UTR, 3′ poly (A) tail, and 3′ enhancer positioned downstream of the EGFP reporter. In addition, an independent SV40 promoter-driven neomycin resistance cassette was used to allow for positive selection of cells that acquired the plasmid irrespective of their ability to activate the Sox2 promoter (Fig. 1).

### Sorting Living Endogenous Sox2-expressing Cervical Cancer Cells Using the pSox2/EGFP System

**Figure 2:**
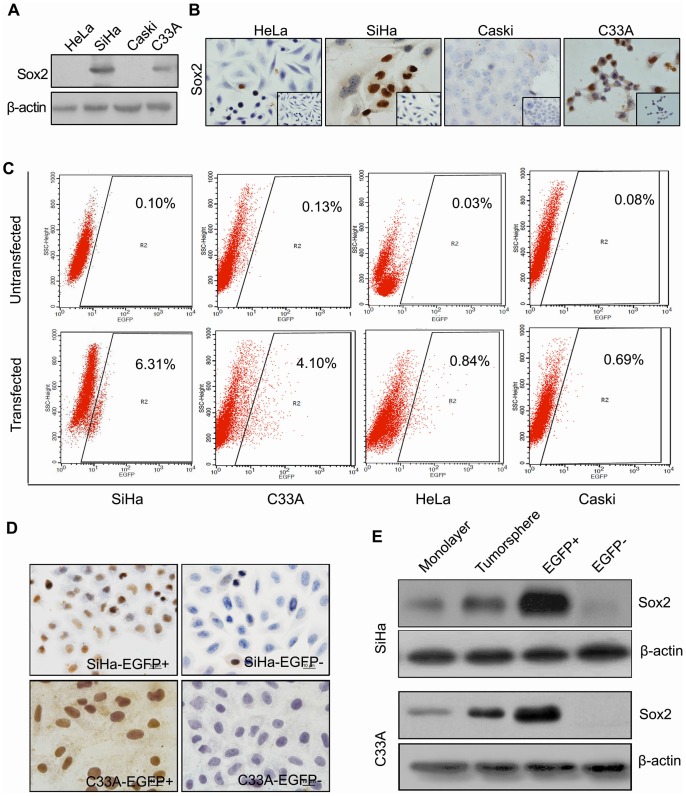
Sorting living cervical cancer cells using the nuclear protein marker Sox2. Sox2 protein expression was detected by western blot (A) and immunohistochemistry (B) in the cervical cancer cell lines HeLa, SiHa, CaSki, and C33A. (C) The abundance of EGFP-positive tumor cells in cervical cancer cell lines transfected with the pSox2/EGFP reporter. (D) Immunohistochemical analysis of EGFP^+^ and EGFP^−^ cells. (E) Sox2 protein expression in a monolayer, tumorsphere cells, and sorted EGFP^+^ and EGFP^−^ cells.

Sox2 expression was characterized in four human cervical cancer cell lines (HeLa, SiHa, CaSki, and C33A) using the pSox/EGFP system as well as western blot and immunocytochemical analysis (Fig. 2A). Sox2 was visible as nuclear staining by immunochemistry in single or clustered SiHa and C33A cells (Fig. 2B). However, Sox2 was not detectable in HeLa or CaSki cells by western blot or immunochemical analysis (Fig. 2A and 2B). When pSox2/EGFP was transiently transfected into four human cervical cancer cell lines, 6.31% and 4.10% of EGFP-positive cells were detectable by FACS in the transfected SiHa and C33A cells, respectively (Fig. 2C). However, only 0.84% and 0.69% of EGFP-positive cells were observable in the transfected HeLa and CaSki cells, respectively (Fig. 2C). EGFP-positive cells comprised less than 0.2% of each untransfected control cervical cancer cell line (Fig. 2C), implying that most EGFP-positive cells in pSox2/EGFP-transfected cervical cancer cells were endogenous Sox-expressing cells. Overall, the results obtained using the pSox2/EGFP system were consistent with the Sox2 expression results from western blot and immunocytochemical analysis in all four cervical cancer cell lines. These results suggest that the pSox2/EGFP plasmid system is suitable for isolating endogenous Sox2-expressing cervical cancer cells. Additionally, because more SiHa and C33A cells endogenously expressed Sox2 compared to HeLa and CaSki cells, the SiHa and C33A cell lines were chosen to evaluate whether endogenous Sox2-expressing cells are CSCs in cervical carcinomas.

To obtain living Sox2-expressing cells, SiHa and C33A cells were transfected with the pSox2/EGFP plasmid. Following selection in culture with 1000 µg/mL G418 for two weeks, all G418-resistant cells were collected and sorted using a FACSAria I; the cells that fell into the <10^th^ and >90^th^ percentiles of EGFP expression were sorted and collected into two separate tubes. The first tube, which contained the cells with the highest EGFP expression, was found to contain more than 90% of all EGFP-expressing cells and was referred to as the EGFP-positive population. The second tube, which contained the cells in the lowest 10% of EGFP expression, only contained EGFP-non-expressing cells and was referred to as the EGFP-negative population (Fig. 2D). Furthermore, western blot and immunocytochemical analysis revealed that only EGFP^+^ SiHa and EGFP^+^ C33A cells expressed Sox2 protein; EGFP^−^ SiHa and EGFP^−^ C33A cells contained no detectable Sox2 protein (Fig. 2E). These results indicate that the pSox2/EGFP system can be used to successfully sort living cells that endogenously express Sox2 from cervical cancer cell lines.

### EGFP-positive Cells have a Higher Capacity for Tumorigenicity than EGFP-negative Cells

**Figure 3:**
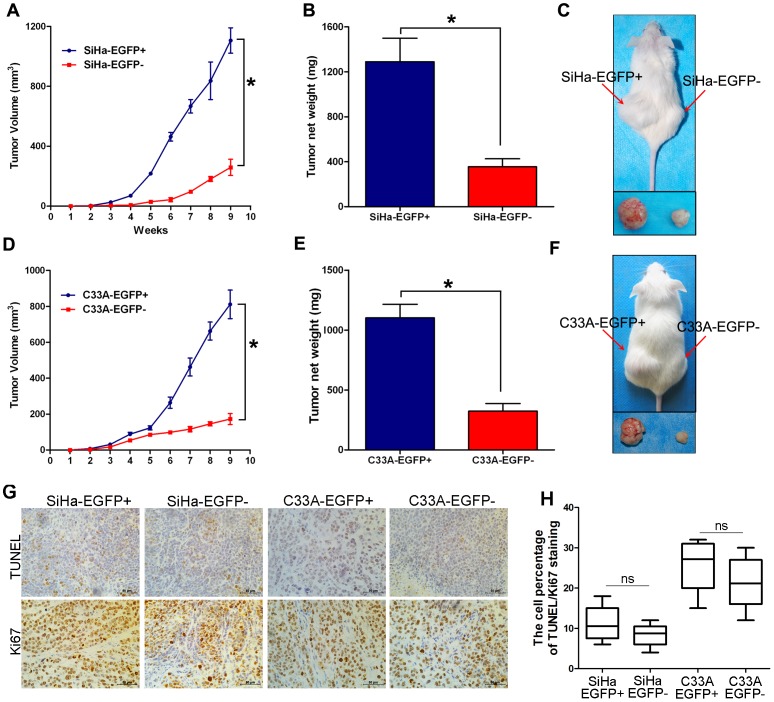
EGFP-positive cells exhibit a greater capacity for tumorigenicity than EGFP-negative cells. Tumor growth curves (A) and tumor weights (B, C) of NOD/SCID mice inoculated with SiHa-EGFP^+^ and SiHa-EGFP^−^ cells (1×10^5^ cells). Tumor growth curves (D) and tumor weights (E, F) of NOD/SCID mice inoculated with C33A-EGFP^+^ and C33A-EGFP^−^ cells (1×10^5^ cells). The apoptosis/proliferation cell rate was evaluated by Tunel assays and Ki67 staining in xenograft (G and H) of EGFP+ and EGFP- cells. Bars = SE. *, p<0.05. ns, p>0.05.

One of the most important characteristics of CSCs is their powerful ability to form tumors. To test the tumor formation ability of the EGFP+ cervical cancer cells, the EGFP-positive and EGFP− cell populations sorted from both the SiHa and C33A cell lines were subcutaneously injected into NOD/SCID mice. When 10^5^ cells were inoculated into NOD/SCID mice, both EGFP+ and EGFP− populations of SiHa and C33A cells formed tumors. However, the tumors formed by the SiHa-EGFP^+^ cells grew much faster (Fig. 3A) and were much larger (Fig. 3B and 3C) than those formed by the SiHa-EGFP^−^ cells (Fig. 3A, B, and C; p<0.05). Similarly, the C33A-EGFP^+^ cells formed tumors that grew much faster and were much larger than those formed by C33A-EGFP^−^ cells (Fig. 3D, E and F; p<0.05). Tumor formation as a function of inoculating different dilutions of EGFP^+^ and EGFP^−^ SiHa and C33A cells is summarized in Table 1. The tumor-initiating frequency of the SiHa-EGFP^+^ cells was 1/402, which was 17.7-fold higher than that of the SiHa-EGFP^−^ cells (1/7114; p<0.001). The tumor-initiating frequency of the C33A-EGFP^+^ cells was 1/3058, which was 23.8-fold higher than that of the C33A-EFGP^−^ cells (1/72,860; p<0.001), suggesting that EGFP-positive SiHa and C33A population contained more CSCs than EGFP-negative population. Furthermore, the apoptosis/proliferation cell rate was evaluated in xenograft (TUNEL/Ki67 staining) to rule out the possibility that decreased tumor growth by EGFP− cells is due to a less cell viability, The TUNEL and Ki67 staining results in the xenograft tissues by SiHa and C33A cells was shown in Fig 3G and summarized in Fig. 3H. There is not any differences in TUNEL and Ki67 positive cell rate in xenograft tissues formed by the Sox2-positive and negative SiHa and C33A cells, suggesting the decreased tumor growth by EGFP− cell was not due to the less cell viability. Taken together, all these results indicate that endogenous Sox2-expressing cervical cancer cells have significantly enhanced tumor formation ability because the EGFP+ SiHa and C33A cells contained more CSCs than EGFP- cells.

**Table 1:** Tumor incidence following xenotransplantation of EGFP^+^ and EGFP^−^ cells in cervical cancer cell lines SiHa and C33A.

Cell line	Sub-population	Incidence				Stem cell frequency	*P* value
		10^5^	10^4^	10^3^	10^2^		
SiHa	EGFP^+^	10/10	10/10	8/10	5/10	1∶402 (1∶812∼1∶200)	1.32e−09
	EGFP^−^	10/10	6/10	4/10	0/10	1∶7114 (1∶14551∼1∶3587)	
C33A	EGFP^+^	10/10	9/10	4/10	1/10	1∶3058 (1∶6313∼1∶1481)	4.01e−11
	EGFP^−^	6/10	3/10	1/10	0/10	1∶72860 (1∶146808∼1∶36160)	

NOTE: Xenotransplantation assay was used to determine the tumor incidence and stem cell frequency of EGFP^+^ and EGFP^−^ cells.

### EGFP-positive Cells Exhibit a Higher Capacity for Self-renewal and Differentiation than EGFP-negative Cells

**Figure 4:**
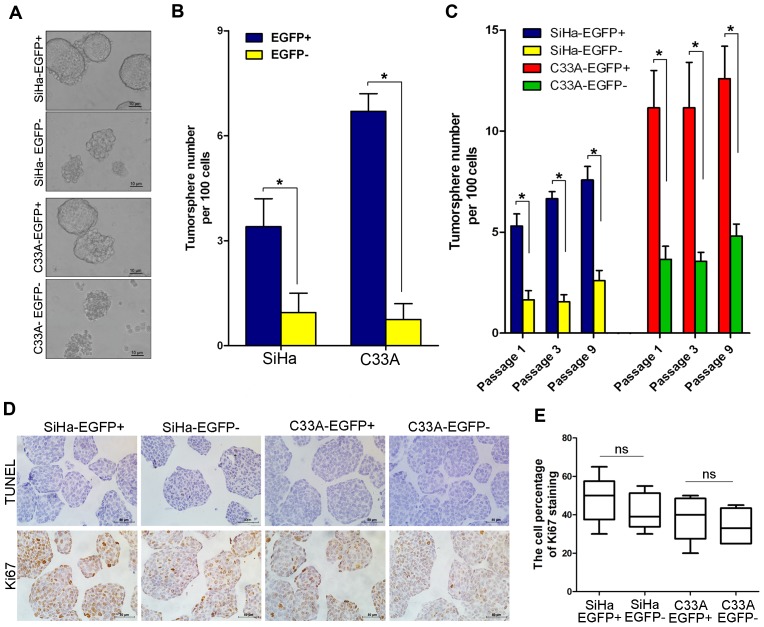
EGFP-positive cells exhibit a greater capacity for self-renewal than EGFP-negative cells. (A, B) Tumorsphere formation assay photographs and the number of tumorspheres formed by SiHa and C33A cells in low-density cultures. (C) Tumorsphere formation assay of EGFP^+^ cells during passaged culture following single-cell sorting by FACS. The apoptosis/proliferation cell rate was evaluated by Tunel assays and Ki67 staining in xenograft (D and E) of EGFP+ and EGFP- cells. Bars = SE. *, p<0.05. ns, p>0.05.

Aside from tumor formation ability, CSCs share two critical properties with stem cells: self-renewal and differentiation. The tumorsphere formation assay is recognized as a classic assay for self-renewal. EGFP-positive and EGFP-negative SiHa and C33A cervical cancer cells were cultured in serum-free medium under conditions that were optimal for growing tumorspheres. As shown in Fig. 4A, EGFP-positive cells isolated from the SiHa and C33A cell lines generated classical tumorspheres, whereas the EGFP-negative cells formed some cell aggregates but did not form tumorspheres. To exclude the effects of cell aggregation, which can occur in low-density cultures, the cells were cultured at a density of 1 cell/well after single-cell sorting by FACS to test their capacity for tumorsphere formation in 96-well plates. Approximately 6% of EGFP-positive SiHa cells formed tumorspheres, which was significantly higher than the percentage of EGFP-negative SiHa cells that formed tumorspheres (approximately 1.6% tumorsphere formation; p<0.05). Similarly, more EGFP-positive C33A cells formed tumorspheres (approximately 12%) compared to EGFP-negative C33A cells (approximately 3%) (Fig. 4B; p<0.05). Furthermore, both EGFP-positive SiHa and C33A cells were able to form tumorspheres in three consecutive passages of tumorsphere cultures, and these cells formed many more tumorspheres than EGFP-negative cells in each culture passage (Fig. 4C; p<0.05). Furthermore, the apoptosis/proliferation cell rate was evaluated in tumorsphere (TUNEL/Ki67 staining) to rule out the possibility that decreased tumor growth by EGFP− cells is due to less cell viability. TUNEL and Ki67 staining results in tumorspheres by SiHa and C33A cells was shown in Fig 4D and summarized in Fig. 4E, in which there is not any differences in TUNEL and Ki67 positive cell rate in the tumorspheres between the Sox2-positive and negative SiHa and C33A cells, suggesting that the decreased tumorsphere formation capacity by EGFP− cells was not due to the less cell viability. Taken together, these findings indicate that endogenous Sox2-expressing SiHa and C33A cells possibly contain more cells that have the self-renewal ability.

**Figure 5:**
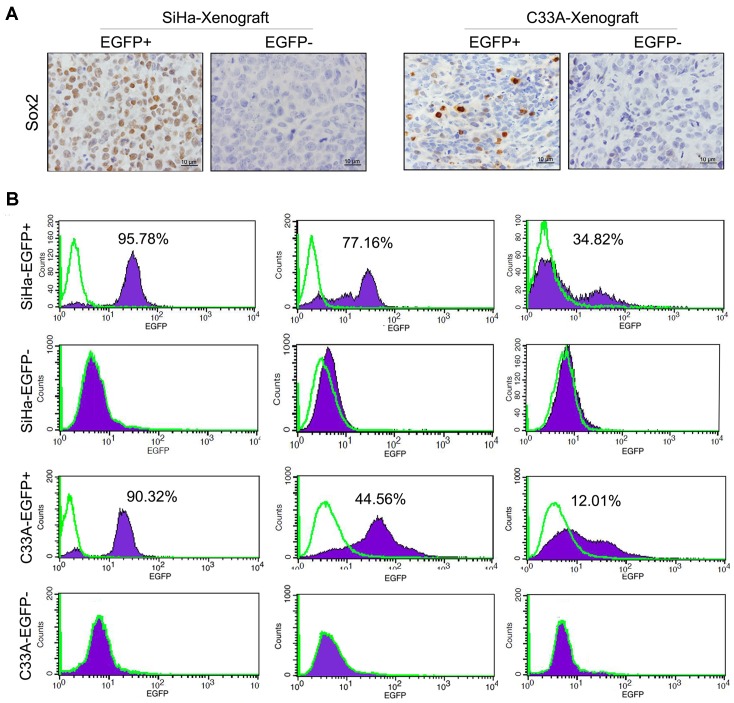
EGFP-positive cells exhibit a greater capacity for differentiation than EGFP-negative cells. (A) Sox2 expression was detected by IHC in xenograft tissues formed by SiHa-EGFP+, SiHa-EGFP−, C33A-EGFP+ and C33A-EGFP− cells. (B) The percentages of EGFP+ cells in the first, second, and third passages in differentiation medium. Bars = SE. *, p<0.05.

In order to identify the self-renewal ability *in vivo*, the serial transplantation assay were performed with 10^2^ of the Sox2 positive and negative SiHa and C33A cells, respectively. The Sox2-positive SiHa and C33A cells could form the tumors in serial three passages of transplantation experiments. However, 10^2^ of the Sox2 negative cells could not form any tumor in the first transplantation experiment. Therefore, the Sox2 positive SiHa and C33A cells have more self-renewal ability than the negative cells *in vivo*. Furthermore, the Sox2 protein was detected by immunohistochemical staining in tumor xenograft tissues formed by EGFP-positive and EGFP-negative SiHa and C33A cells (Fig 5A). Both the Sox2-positive and Sox2-negative cells were found in the tumor xenografts formed by the EGFP-positive cells. However, only Sox2-negative cells could be detected in the tumor tissues formed by the Sox2-negative cervical cancer cells. These results further supported that the Sox2-positive SiHa and C33A cells not only have the self-renewal ability to reproduce the Sox2-positive cells, but also have the differentiation ability to produce the Sox2-negative cells *in vivo*.

When the EGFP-positive SiHa and C33A cells were cultured in DMEM containing 10% FBS, the percentage of EGFP-positive SiHa cells decreased from 95.78% in the first passage to 77.16% in the second passage and 34.82% in the third passage. Similarly, the percentage of EGFP-positive C33A cells decreased from 90.32% in the first passage to 44.56% and 12.01% in the second and third passages in differentiation media, respectively (Fig. 5B). Additionally, EGFP-negative SiHa and C33A cells could only generate EGFP-negative cells and could not produce any EGFP-positive cells (Fig. 5B). These results indicate that only endogenous Sox2-expressing cervical cancer cells have differentiation ability in vitro.

### EGFP-positive Cervical Cancer Cells Express More Stem Cell-related Proteins than EGFP-negative Cells

**Figure 6:**
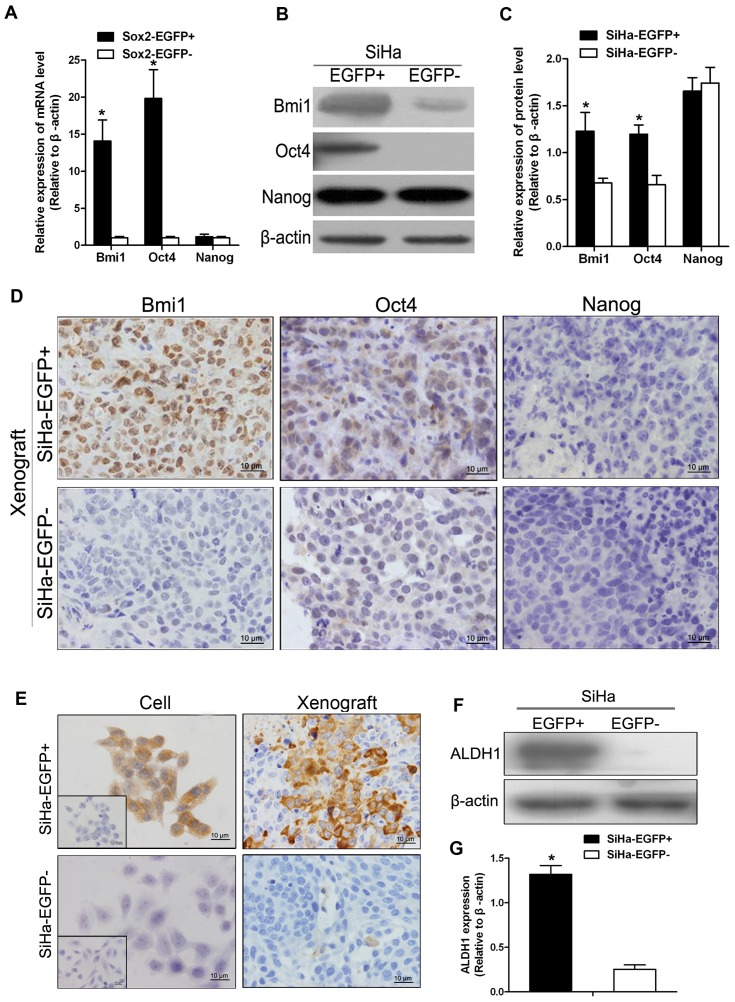
Distinct molecular and biological properties of Sox2-positive and Sox2-negative cells. (A) Differential expression of several stem cell-related genes in SiHa-EGFP^+^ and SiHa-EGFP^−^ fractions validated by qPCR. (B) Detection of stem cell-related factors in sorted SiHa-EGFP^+^ and SiHa-EGFP^−^ cells by western blot. (C) Semi-quantitative analysis of stem cell-related factors relative to β-actin. (D) Stem cell-related gene expression in tumor xenografts was detected by immunohistochemistry. ALDH1 was detected by immunohistochemistry (E), western blot (F), and semi-quantitative analysis (G) in SiHa-EGFP^+^ and SiHa-EGFP^−^ tumors. β-actin was used as the loading control for RT-PCR and western blotting. Error bars represent S.D. (n = 3). * p<0.05.

Sox2, OCT4, Bmi1, and Nanog are recognized as stem cell self-renewal-related nuclear transcription factors. We compared the expression of these transcription factors in EGFP-positive cells, EGFP-negative cells, and tumor xenografts. As shown in Figure 6, real-time PCR (Fig. 6A), western blot analysis (Fig. 6B and C), and immunohistochemical analysis (Fig. 6D) revealed that EGFP-positive SiHa cells and the tumors formed by these cells expressed higher levels of the Bmi1, Oct4, and Sox2 proteins than EGFP-negative SiHa cells at both the transcriptional level (Fig. 6A) and the translational level (Fig. 6B, C and D). However, Nanog protein expression was unchanged.

Furthermore, in recent years, a growing number of studies have indicated that ALDH-positive cells represent CSCs in many somatic tumors [23]. In the present study, immunochemical staining (Fig. 6E, left) and western blot assays (Fig. 6F and G) demonstrated that EGFP-positive cells expressed ALDH, whereas EGFP-negative cells did not. Immunohistochemical analysis also revealed that the tumor tissues formed by EGFP-positive SiHa cells, not those formed by EGFP-negative SiHa cells, were stained by ALDH1 antibody (Fig. 6E, right). In C33A cells, the stem cell related genes Bmi1, Oct4 and Nanog were also detected by western blot. The result showed that C33A-EGFP+ subpopulation cells expressed much stronger than C33A-EGFP− cells, expect Nanog, which was similar to SiHa cells (Figure S1, A and B). Additionally, ALDH1 was detectable in EGFP+ cells but not in EGFP− cells by western blot and IHC (Figure S1, A–C). Taken together, these results suggest that EGFP-positive cervical cancer cells also express some stem cell-related factors, such as OCT4, Bmi1, and ALDH1.

### EGFP-positive Cervical Cancer Cells Exhibited More EMT Features than EGFP-negative Cells

**Figure 7:**
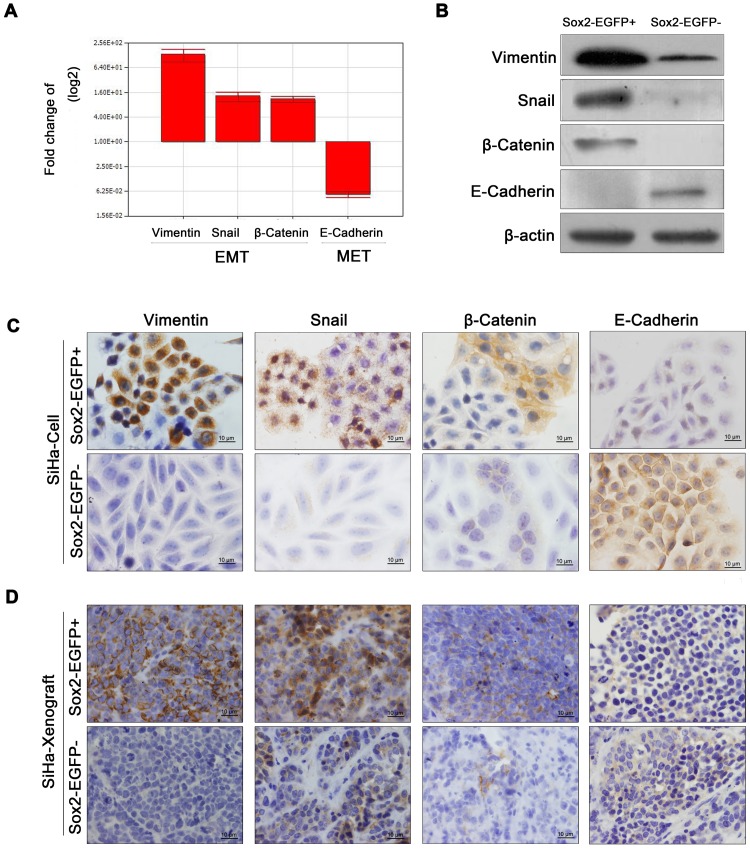
Sox2-positive cells exhibit more EMT features but do not show altered proliferation *in vitro*. (A) Real-time PCR analysis of the mRNA levels of various EMT-related genes in SiHa-EGFP^+^ and SiHa-EGFP^−^ cells. EMT-related gene expression was measured by western blot (B) and immunochemistry (C) in SiHa-EGFP^+^ and SiHa-EGFP^−^ cells. (D) Immunochemistry for Sox2 and EMT-related genes in tumor xenografts. Bars = SE. *, p<0.05.

EMT induction has been reported to generate cells with stem-like properties [24], and CSCs share some properties with cells undergoing EMT [25], [26]. Here, we assessed the status of EMT markers in Sox2-positive cells, Sox2-negative cells, and tumor tissues formed by these cells (Fig. 7). In SiHa-EGFP^+^ cells and tumor tissues, real-time PCR demonstrated that mesenchyme proteins such as vimentin, snail, and β-catenin were markedly upregulated at the mRNA level (Fig. 7A). Similarly, Western blot (Fig. 7B), immunocytochemical (Fig. 7C), and immunohistochemical analyses (Fig. 7D) demonstrated that they were also upregulated at the protein level. In contrast, E-cadherin, a well-known epithelial marker, was downregulated in EGFP-positive SiHa cells and upregulated in EGFP-negative cells and tumor xenografts (Fig. 7A–D). We also found that the EMT-related genes, vimentin and β-catenin, were expressed at a higher level in EGFP-positive C33A cells than that in EGFP-negative cells by Western blot and immunocytochemical staining. The epithelial marker E-cadherin was detectable in C33A-EGFP− cells, but not in C33A-EGFP+ cells. However, the Snail protein was not expressed in both EGFP-positive and EGFP-negative cells. (Figure S2). Collectively, these results indicate that cells that endogenously express Sox2 possess the capacities of self-renewal and differentiation as well as tumorigenicity and the characteristics of cells undergoing EMT. Therefore, the cells that endogenously express Sox2 that were isolated from the SiHa and C33A cell lines are CSCs in cervical carcinomas.

## Discussion

Although CSCs have been isolated and identified in many somatic cancers, only a few somatic CSC markers, such as CD44+/CD24^−/low^ for breast cancer, have been extensively accepted. To date, most studies exploring CSC isolation have depended primarily on the use of a variety of cell surface markers [27]. However, most cell surface markers are absent or not stably expressed on somatic tumor cells; as such, it is difficult to identify both CSCs and diversity among CSCs in somatic cancer [28]. In recent years, increasing efforts have been focused on the identification of cytoplasmic or nuclear markers of CSCs, and ALDH activity has been recognized as a cytoplasmic marker for many somatic CSCs [29], [30]. Additionally, OCT4 has been shown to be a nuclear marker of osteosarcoma stem cells [31]. Our previous studies have indicated that Sox2 is a potential nuclear marker of cervical carcinoma [14], [15]. In the present study, we tested whether cervical CSCs could be isolated using the nuclear protein Sox2.

To isolate the living subpopulation of cells expressing Sox2, we constructed the pSox2/EGFP plasmid, which contains the entire 11.5-kb human Sox2 promoter sequence upstream of an EGFP reporter with the Sox2 3′UTR and an enhancer sequence downstream of the EGFP reporter. After transfection with this plasmid, Sox2 transcription factors in endogenous Sox2-expressing cells transactivated the Sox2 transcriptional elements in the plasmid, causing the Sox2-expressing cells to express EGFP. Because Sox2-negative cells contain no Sox2 transcription factors, the pSox2/EGFP plasmid could not be driven to express EGFP following transfection into these cells. Therefore, we were easily able to isolate Sox2-expressing and Sox2-non-expressing cells based on their EGFP-positive or EGFP-negative status using FACS after transfection with the reporter plasmid.

Although the relationship between Sox2 expression and possible CSCs population was identified from breast cancer [32], lung cancer [33], ovarian cancer [34], as well as cervical cancer [14], this is the first study to to isolate and identify CSCs in somatic carcinoma based on the endogenous Sox2 nuclear protein expression. Sox2-positive cells isolated from the cervical cancer cell lines SiHa and C33A were found to exhibit self-renewal, differentiation, and tumor initiating properties, which are major characteristics of CSCs. Furthermore, Sox2-positive SiHa and C33A cells expressed increased levels of the stemness-related factors Oct4 and Bmi1 as well as the putative CSC marker ALDH1. More significantly, Sox2-positive cells retained intrinsically mesenchymal features, expressing antigens commonly associated with mesenchymal stem cells such as vimentin, snail and β-catenin. Therefore, CSCs that express the endogenous nuclear protein Sox2 have all the properties of CSCs, similar to those identified based on cell surface markers. Recently, CD49f was found as a possible surface marker for cervical cancer stem cells [35]. The CD49f was expressed at the much higher level in EGFP+ than that in EGFP− SiHa and C33A cells, respectively, suggesting both Sox2 and CD49f are the appropriate marker for cervical cancer stem cells (Figure S3). Furthermore, further experiments should be designed to figure out which is the best cervical cancer stem cell population isolated by either Sox2 or CD49f or by both.

The pSox2/EGFP isolation system described here awaits further improvements. The plasmid construct contains a large segment of DNA (approximately 16 kb) containing Sox2 transcriptional elements. Therefore, this transfection system is not optimal. It is necessary to analyze the Sox2 transcription elements to define the shortest segments required to allow the development of a lentivirus or retrovirus system that will improve the transfection efficiency. Using such a system, it may be possible to isolate Sox2-positive cells from low-expressing cancer cell lines, such as HeLa and CaSki cells, or from primary cancer cells obtained from fresh cancer tissues.

In conclusion, we first isolated CSCs from cervical cancer cell lines using a functional nuclear marker, Sox2. Sox2-expressing cervical cancer cells shared all the properties of CSCs. This study suggested that the nuclear marker Sox2 may serve as a feasible alternative to surface markers for the isolation of CSCs from somatic carcinomas.

## Supporting Information

Figure S1 (A) Differential expression of several stem cell-related genes and ALDH1 in C33A-EGFP+ and C33A-EGFP− fractions validated by western blot. (B) Semi-quantitative analysis of stem cell-related factors and ALDH1 relative to β-actin. (C) ALDH1 was detected by immunohistochemistry in C33A-EGFP+ and C33A-EGFP− cells. Error bars represent S.D. (n = 3). * p<0.05.
(TIF)

Figure S2 (A) Western blot analysis of the protein levels of various EMT-related genes in C33A-EGFP+ and C33A-EGFP− cells. (B) Semi-quantitative analysis of EMT-related factors relative to β-actin. (C) Immunochemistry for EMT-related genes in C33A-EGFP+ and C33A-EGFP− cells. Bars = SE. *, p<0.05.
(TIF)

Figure S3 CD49f expression was detected by FACS in EGFP^+^ and EGFP^−^ cells of SiHa and C33A.
(TIF)

## References

[1] Bonnet D, Dick JE (1997) Human acute myeloid leukemia is organized as a hierarchy that originates from a primitive hematopoietic cell. Nat Med 3: 730–737. 9212098 10.1038/nm0797-730

[2] Al-Hajj M, Wicha MS, Benito-Hernandez A, Morrison SJ, Clarke MF (2003) Prospective identification of tumorigenic breast cancer cells. Proc Natl Acad Sci U S A 100: 3983–3988. 12629218 10.1073/pnas.0530291100 PMC153034

[3] Singh SK, Hawkins C, Clarke ID, Squire JA, Bayani J, et al (2004) Identification of human brain tumour initiating cells. Nature 432: 396–401. 15549107 10.1038/nature03128

[4] Collins AT, Berry PA, Hyde C, Stower MJ, Maitland NJ (2005) Prospective identification of tumorigenic prostate cancer stem cells. Cancer Res 65: 10946–10951. 16322242 10.1158/0008-5472.CAN-05-2018

[5] Fang D, Nguyen TK, Leishear K, Finko R, Kulp AN, et al (2005) A tumorigenic subpopulation with stem cell properties in melanomas. Cancer Res 65: 9328–9337. 16230395 10.1158/0008-5472.CAN-05-1343

[6] Matsui W, Huff CA, Wang Q, Malehorn MT, Barber J, et al (2004) Characterization of clonogenic multiple myeloma cells. Blood 103: 2332–2336. 14630803 10.1182/blood-2003-09-3064 PMC3311914

[7] O'Brien CA, Pollett A, Gallinger S, Dick JE (2007) A human colon cancer cell capable of initiating tumour growth in immunodeficient mice. Nature 445: 106–110. 17122772 10.1038/nature05372

[8] Ricci-Vitiani L, Lombardi DG, Pilozzi E, Biffoni M, Todaro M, et al (2007) Identification and expansion of human colon-cancer-initiating cells. Nature 445: 111–115. 17122771 10.1038/nature05384

[9] Li C, Heidt DG, Dalerba P, Burant CF, Zhang L, et al (2007) Identification of pancreatic cancer stem cells. Cancer Res 67: 1030–1037. 17283135 10.1158/0008-5472.CAN-06-2030

[10] Prince ME, Sivanandan R, Kaczorowski A, Wolf GT, Kaplan MJ, et al (2007) Identification of a subpopulation of cells with cancer stem cell properties in head and neck squamous cell carcinoma. Proc Natl Acad Sci U S A 104: 973–978. 17210912 10.1073/pnas.0610117104 PMC1783424

[11] Madka V, Rao CV (2011) Cancer stem cell markers as potential targets for epithelial cancers. Indian J Exp Biol 49: 826–835. 22126013

[12] O'Brien CA, Kreso A, Ryan P, Hermans KG, Gibson L, et al (2012) ID1 and ID3 Regulate the Self-Renewal Capacity of Human Colon Cancer-Initiating Cells through p21. Cancer Cell 21: 777–792. 22698403 10.1016/j.ccr.2012.04.036

[13] van den Hoogen C, van der Horst G, Cheung H, Buijs JT, Lippitt JM, et al (2010) High aldehyde dehydrogenase activity identifies tumor-initiating and metastasis-initiating cells in human prostate cancer. Cancer Res 70: 5163–5173. 20516116 10.1158/0008-5472.CAN-09-3806

[14] Ji J, Zheng PS (2010) Expression of Sox2 in human cervical carcinogenesis. Hum Pathol 41: 1438–1447. 20709360 10.1016/j.humpath.2009.11.021

[15] Xu R, Yang WT, Zheng PS (2013) Coexpression of B-lymphoma Moloney murine leukemia virus insertion region-1 and sex-determining region of Y chromosome-related high mobility group box-2 in cervical carcinogenesis. Hum Pathol 44: 208–217. 22863087 10.1016/j.humpath.2012.02.020

[16] Boyer LA, Lee TI, Cole MF, Johnstone SE, Levine SS, et al (2005) Core transcriptional regulatory circuitry in human embryonic stem cells. Cell 122: 947–956. 16153702 10.1016/j.cell.2005.08.020 PMC3006442

[17] Bani-Yaghoub M, Tremblay RG, Lei JX, Zhang D, Zurakowski B, et al (2006) Role of Sox2 in the development of the mouse neocortex. Dev Biol 295: 52–66. 16631155 10.1016/j.ydbio.2006.03.007

[18] Episkopou V (2005) SOX2 functions in adult neural stem cells. Trends Neurosci 28: 219–221. 15866195 10.1016/j.tins.2005.03.003

[19] Leis O, Eguiara A, Lopez-Arribillaga E, Alberdi MJ, Hernandez-Garcia S, et al. (2011) Sox2 expression in breast tumours and activation in breast cancer stem cells. Oncogene. 10.1038/onc.2011.338 21822303

[20] Gangemi RM, Griffero F, Marubbi D, Perera M, Capra MC, et al (2009) SOX2 silencing in glioblastoma tumor-initiating cells causes stop of proliferation and loss of tumorigenicity. Stem Cells 27: 40–48. 18948646 10.1634/stemcells.2008-0493

[21] Yang WT, Zheng PS (2012) Kruppel-like factor 4 functions as a tumor suppressor in cervical carcinoma. Cancer 118: 3691–3702. 22170594 10.1002/cncr.26698

[22] Giakoumopoulos M, Siegfried LM, Dambaeva SV, Garthwaite MA, Glennon MC, et al (2010) Placental-derived mesenchyme influences chorionic gonadotropin and progesterone secretion of human embryonic stem cell-derived trophoblasts. Reprod Sci 17: 798–808. 20601539 10.1177/1933719110371853 PMC3065864

[23] Sun S, Wang Z (2010) ALDH high adenoid cystic carcinoma cells display cancer stem cell properties and are responsible for mediating metastasis. Biochem Biophys Res Commun 396: 843–848. 20450887 10.1016/j.bbrc.2010.04.170

[24] Shimono Y, Zabala M, Cho RW, Lobo N, Dalerba P, et al (2009) Downregulation of miRNA-200c links breast cancer stem cells with normal stem cells. Cell 138: 592–603. 19665978 10.1016/j.cell.2009.07.011 PMC2731699

[25] Ocana OH, Corcoles R, Fabra A, Moreno-Bueno G, Acloque H, et al (2012) Metastatic colonization requires the repression of the epithelial-mesenchymal transition inducer Prrx1. Cancer Cell 22: 709–724. 23201163 10.1016/j.ccr.2012.10.012

[26] Tsai JH, Donaher JL, Murphy DA, Chau S, Yang J (2012) Spatiotemporal regulation of epithelial-mesenchymal transition is essential for squamous cell carcinoma metastasis. Cancer Cell 22: 725–736. 23201165 10.1016/j.ccr.2012.09.022 PMC3522773

[27] Sakashita H, Ieta K, Haraguchi N, Inoue Y, Yoshizawa Y, et al (2007) [Cancer stem cell]. Gan To Kagaku Ryoho 34: 1721–1729. 18030004

[28] Yang YM, Chang JW (2008) Current status and issues in cancer stem cell study. Cancer Invest 26: 741–755. 18608212 10.1080/07357900801901856

[29] Huang EH, Hynes MJ, Zhang T, Ginestier C, Dontu G, et al (2009) Aldehyde dehydrogenase 1 is a marker for normal and malignant human colonic stem cells (SC) and tracks SC overpopulation during colon tumorigenesis. Cancer Res 69: 3382–3389. 19336570 10.1158/0008-5472.CAN-08-4418 PMC2789401

[30] Tomuleasa C, Mosteanu O, Susman S, Cristea V (2011) ALDH as a tumor marker for pancreatic cancer. J Gastrointestin Liver Dis 20: 443–444; author reply 444. 22187714

[31] Levings PP, McGarry SV, Currie TP, Nickerson DM, McClellan S, et al (2009) Expression of an exogenous human Oct-4 promoter identifies tumor-initiating cells in osteosarcoma. Cancer Res 69: 5648–5655. 19584295 10.1158/0008-5472.CAN-08-3580 PMC2841219

[32] Leis O, Eguiara A, Lopez-Arribillaga E, Alberdi MJ, Hernandez-Garcia S, et al (2012) Sox2 expression in breast tumours and activation in breast cancer stem cells. Oncogene 31: 1354–1365. 21822303 10.1038/onc.2011.338

[33] Xiang R, Liao D, Cheng T, Zhou H, Shi Q, et al (2011) Downregulation of transcription factor SOX2 in cancer stem cells suppresses growth and metastasis of lung cancer. Br J Cancer 104: 1410–1417. 21468047 10.1038/bjc.2011.94 PMC3101944

[34] Bareiss PM, Paczulla A, Wang H, Schairer R, Wiehr S, et al (2013) SOX2 expression associates with stem cell state in human ovarian carcinoma. Cancer Res 73: 5544–5555. 23867475 10.1158/0008-5472.CAN-12-4177

[35] Lopez J, Poitevin A, Mendoza-Martinez V, Perez-Plasencia C, Garcia-Carranca A (2012) Cancer-initiating cells derived from established cervical cell lines exhibit stem-cell markers and increased radioresistance. BMC Cancer 12: 48. 22284662 10.1186/1471-2407-12-48 PMC3299592

