# Supplementary figures and images for: Cervical Cancer Cells with Positive Sox2 Expression Exhibit the Properties of Cancer Stem Cells
Source: PLoS One. 2014 Jan 28;9(1):e87092. doi: 10.1371/journal.pone.0087092 (PMC3904967; doi:10.1371/journal.pone.0087092)

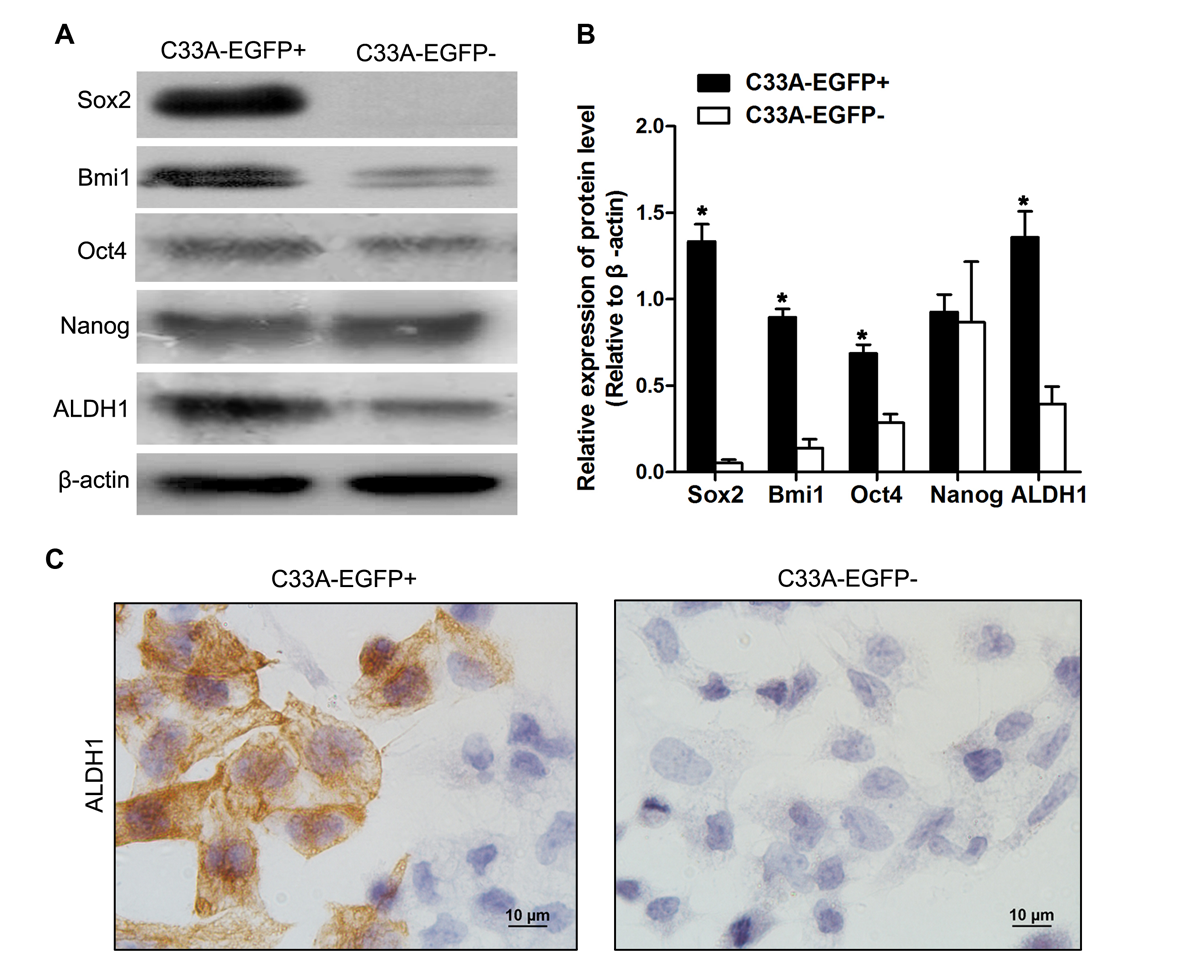

Supplement: Figure S1 — (A) Differential expression of several stem cell-related genes and ALDH1 in C33A-EGFP+ and C33A-EGFP− fractions validated by western blot. (B) Semi-quantitative analysis of stem cell-related factors and ALDH1 relative to β-actin. (C) ALDH1 was detected by immunohistochemistry in C33A-EGFP+ and C33A-EGFP− cells. Error bars represent S.D. (n = 3). * p<0.05. (TIF) [file pone.0087092.s001.tif]

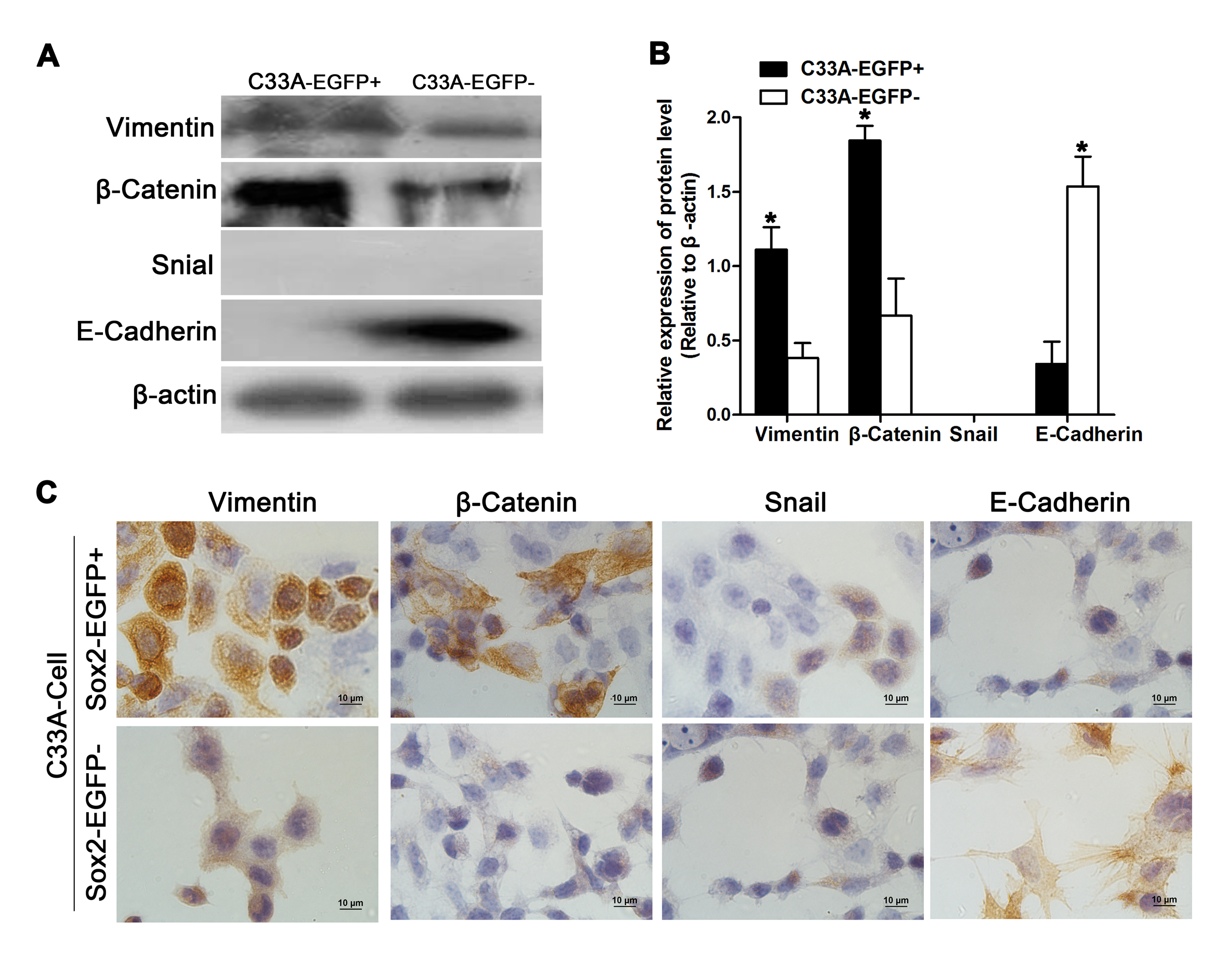

Supplement: Figure S2 — (A) Western blot analysis of the protein levels of various EMT-related genes in C33A-EGFP+ and C33A-EGFP− cells. (B) Semi-quantitative analysis of EMT-related factors relative to β-actin. (C) Immunochemistry for EMT-related genes in C33A-EGFP+ and C33A-EGFP− cells. Bars = SE. *, p<0.05. (TIF) [file pone.0087092.s002.tif]

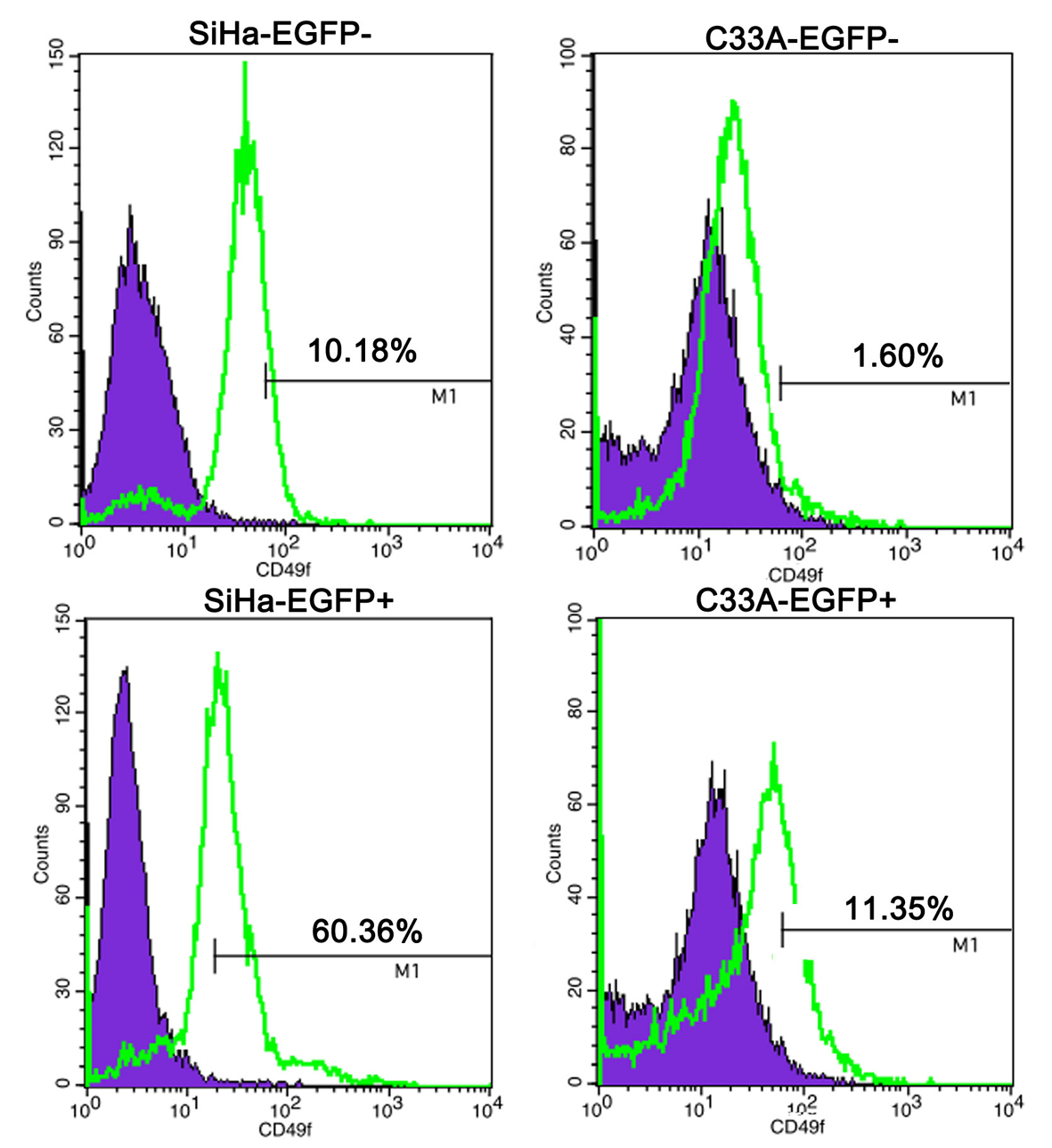

Supplement: Figure S3 — CD49f expression was detected by FACS in EGFP+ and EGFP− cells of SiHa and C33A. (TIF) [file pone.0087092.s003.tif]
